# The Development of BTK Inhibitors: A Five-Year Update

**DOI:** 10.3390/molecules26237411

**Published:** 2021-12-06

**Authors:** Bruno Tasso, Andrea Spallarossa, Eleonora Russo, Chiara Brullo

**Affiliations:** Department of Pharmacy, University of Genova, Viale Benedetto XV 3, 16132 Genova, Italy; tasso@difar.unige.it (B.T.); andrea.spallarossa@unige.it (A.S.); russo@difar.unige.it (E.R.)

**Keywords:** Bruton’s kinase, (ir)reversible inhibitors, B-cell malignancies, autoimmune diseases, medicinal chemistry, small molecules

## Abstract

Bruton’s tyrosine kinase (BTK) represented, in the past ten years, an important target for the development of new therapeutic agents that could be useful for cancer and autoimmune disorders. To date, five compounds, able to block BTK in an irreversible manner, have been launched in the market, whereas many reversible BTK inhibitors (BTKIs), with reduced side effects that are more useful for long-term administration in autoimmune disorders, are under clinical investigation. Despite the presence in the literature of many articles and reviews, studies on BTK function and BTKIs are of great interest for pharmaceutical companies as well as academia. This review is focused on compounds that have appeared in the literature from 2017 that are able to block BTK in an irreversible or reversible manner; also, new promising tunable irreversible inhibitors, as well as PROTAC molecules, have been reported. This summary could improve the knowledge of the chemical diversity of BTKIs and provide information for future studies, particularly from the medicinal chemistry point of view. Data reported here are collected from different databases (Scifinder, Web of Science, Scopus, Google Scholar, and Pubmed) using “BTK” and “BTK inhibitors” as keywords.

## 1. Introduction

Bruton’s tyrosine kinase (BTK) is a cytoplasmatic kinase belonging to the Tec family and is expressed by hematopoietic and plasma cells that regulate different signals, including the PI3K, MAPK, and NF-κB pathways [1,2]. In the last twenty years, the discovery that many types of cancer can be characterized by the hyperactivation/hyperregulation of different tyrosine kinases (TK) has led to their consideration as new and interesting therapeutic targets in cancer therapy. Starting from Imatinib (a Bcr-Abl tyrosine kinase inhibitor approved in 2001 for chronic myeloid leukemia), a plethora of TK inhibitors were launched on the market (23 FDA-approved TKIs from 2018). Due to the oral bioavailability of TK inhibitors, as well as the benefits of targeted therapy, today this type of compounds represents the first/second-line therapy for the treatment of different cancers [3].

BTK is expressed in all hematopoietic cells (particularly B-lymphocytes) except for T-lymphocytes and terminally differentiated plasma cells. In fact, it is an essential component of different B-cell receptor (BCR) signal pathways that regulate the differentiation of antibody-producing plasma cells, as well as cell activation, proliferation, and survival [4]. In addition, BTK plays a crucial role in the secretion of pro-inflammatory cytokines, as well as degranulation and histamine release [1,5,6].

Therefore, BTK inhibition causes a block of different down-stream cell signalling pathways strictly related to the development of B-cell malignancies, as well as autoimmune diseases [7]. Since the BTK structure and function were well defined in 1993 by Vetrie et al. [8], BTK inhibitors (BTKIs) have been studied for the treatment of different haematological disorders (particularly of B-cell malignancies) [9] and of many autoimmune diseases; consequently, many investigations by industry and academia have been performed. In fact, BTKIs could have some advantages over biological compounds, being less likely to trigger antibody responses and consequent allergic reactions [1].

## 2. BTKIs: Their Classification

BTK consists of 659 amino acids and five domains from the N-terminus to the C-terminus: the pleckstrin homology (PH) domain, the proline-rich TEC homology (TH) domain, the SRC homology (SH) domains (named SH3 and SH2), and finally the catalytic domain [7,10]. The PH domain mediates protein–phospholipid and protein–protein interactions, whereas and the TH domain contains a zinc finger motif important for protein activity and stability. SH2 and SH3 domains contain the autophosphorylation site Tyr223, whereas the catalytic domain contains two phosphorylation sites (Tyr551 and Cys481) targeted by irreversible inhibitors. These structural characteristics are fundamental for the design of different type of BTKIs.

On the basis of their mechanisms of action and binding modes, BTKIs can be classified into two types: (i) irreversible inhibitors, characterized by a Michael acceptor moiety able to form a covalent bond with the conserved Cys481 residue in the ATP binding site, or (ii) reversible inhibitors that bind to a specific pocket in the SH3 domain through weak, reversible interactions (e.g., hydrogen bonds or hydrophobic interactions), causing an inactive conformation of the enzyme. More recently, a new hybrid BTKIs emerged: BTK is able to bind in a reversible covalent manner, forming reversible covalent bonds with Cys481 residue and temporarily inactivating the enzyme. Particularly, this class of inhibitors is highly potent and selective, showing prolonged, tunable residence times and fewer off-target effects, combining the advantages of the covalent and non-covalent inhibitors. In addition, more recently some PROTAC (Proteolysis-targeting chimera) derivatives have been developed [11,12].

Up until now, only irreversible BTKIs have been in the market, whereas in the past ten years a lot of reversible compounds have been patented [4,13] due to their lower toxicity in respect to irreversible derivatives. They are currently under clinical investigation for long-term drug administration in autoimmune disorders, particularly rheumatoid arthritis (RA), primary and secondary progressive multiple sclerosis (MS), systemic lupus erythematosus (SLE), Sjögren syndrome, uticaria, pemphigus, and many others. In fact, covalent and irreversible inhibitions increase the risk of off-target reactivity to biomolecules, resulting in immunotoxicity, mutagenicity, and hepatotoxicity. In addition, because it has been demonstrated that both B-cells and cells of the myeloid lineage are important in the development of MS, an increased interest in developing BTKIs for MS treatment has emerged [14,15]. 

## 3. Irreversible BTKIs on the Market

Ibrutinib (Table 1, Imbruvica) was the first BTKI approved by the FDA in 2013 for the treatment of chronic lymphocytic leukemia (CLL), small lymphocytic lymphoma (SLL), Waldenström’s macroglobulinemia (WM), marginal zone lymphoma (MZL), and relapsed/refractory mantle cell lymphoma (MCL); it was also approved in 2017 for chronic graft-versus-host disease (cGVHD) patients. Its approval has had epoch-making significance and it became one of the five top-selling medicines in the world [10]. More recently, Acalabrutinib and Zanubrutinib (Table 1, Calquence and Brukinsa, respectively), new BTKIs with reduced off-target effects, were launched in the market in 2017 and 2019, respectively, for CLL, SLL, and MCL [6]. In addition, Tirabrutinib (Table 1, Velexbru) is currently registered only in Japan by the Pharmaceuticals and Medical Devices Agency (PMDA) for the treatment of recurrent or refractory primary central nervous system lymphoma and has also received supplemental approval for WM and lymphoplasmacytic lymphoma [16,17]. In December 2020, Orelabrutinib (Table 1) received its first approval in China for the treatment of patients with MCL, CLL, and SLL who have received at least one treatment in the past. The clinical development of Orelabrutinib for various indications is underway in the USA and China [18].

From 2020, approved BTKIs are being studied for COVID-19 patient treatments (NCT04375397, NCT04665115, NCT04497948, NCT04380688, NCT04346199, and NCT04382586), but results are not available at the moment. Data from the CALAVI phase II trials (NCT04497948) for Acalabrutinib in hospitalized COVID-19 patients did not meet the primary efficacy endpoints and, on the basis of these results, this study has been prematurely terminated [19,20].

Unfortunately, Ibrutinib and all irreversible BTK inhibitors also inhibit other kinases that possess Cys481-like residues, including intracellular (Tec, Itk, and Blk) and receptor (e.g., epidermal growth factor receptors (EGFR)) tyrosine kinases. This lack of selectivity is responsible of many off-target side effects (i.e., skin and dermatological problems, allergic reactions, fever, lymphadenopathy, edema, albuminuria, diarrhea, bleeding, infection, headaches, and atrial fibrillation) [6,21].

In addition, the onset of resistant mutants (especially to Ibrutinib) has reduced their use. In particular, the isosteric replacement of Cys481 with a serine residue decreases the activity of the major part of irreversible BTKIs. Furthermore, other site mutations involving both Cys481 (e.g., C481R, C481F, and C481Y) and the gatekeeper residue Thr474 (T474I, T474S, and T474M) have been recently evidenced. In this regard, reversible inhibitors that do not interact with Cys481 could inhibit C481R, T474I, and T474M mutants, as well as representing an interesting therapeutic option [22].

Moreover, to date, the use of reversible inhibitors seems to be more effective in treating autoimmune diseases such as RA, different types of MS, cGVHD, and SLE.

## 4. BTKIs in Clinical and Pre-Clinical Evaluations

### 4.1. Irreversible BTKIs

The previous cited approved BTKIs belong to the first class of BTKIs, as well as the major part of the compounds under clinical investigation (Table 2) as recently reported by Liu [23]. All derivatives (with the exception of Elsubrutinib) inhibit BTKs with IC_50_ values in the low nanomolar range.

**Figure 1:**
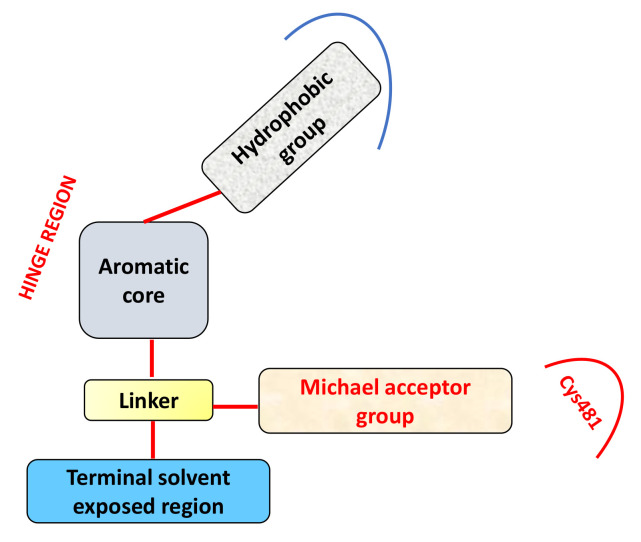
Schematic representation of irreversible BTKIs.

It has been reported that the pharmacophore model of irreversible BTKIs consist in four principal components: (i) a large hydrophobic group, (ii) an aromatic heterocyclic nucleus, (iii) a linker, and (iv) a warhead terminal group. The aromatic core with a hydrogen bond donor and acceptor occupies the BTK hinge region, whereas the large hydrophobic group that extends to the hydrophobic pockets adjacent to the hinge region is responsible of different hydrophobic interactions. The linker connects the aromatic core with the warhead or terminal group able to form a covalent bond with Cys481 and often also with a terminal solvent exposed region (Figure 1) [4,23,24,25,26,27]. Regarding the aromatic core, in some cases, compounds are characterized by a single aromatic ring (particularly a substituted pyrimidine ring as in Spebrutinib, Evobrutinib, and Remibrutinib, Table 2) or a different bicyclic structure (Olmutinib, Elsubrutinib, Tolebrutinib, Branebrutinib, and Rilzabrutinib, Table 2). 

**Figure 2:**
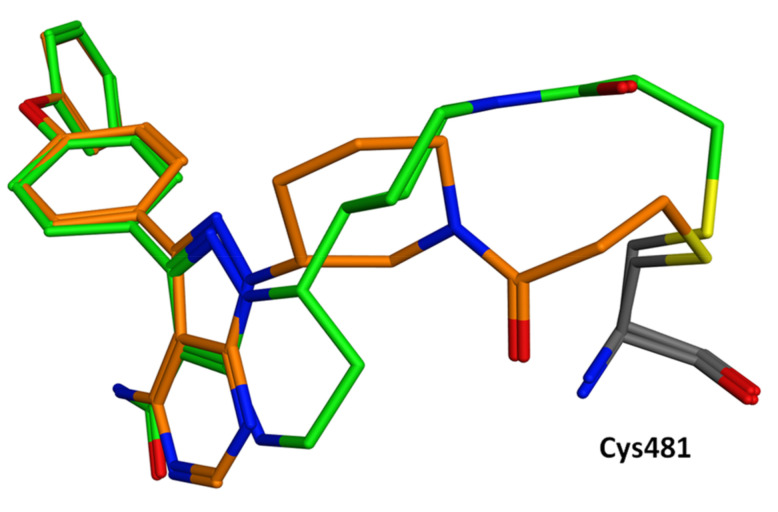
Superposition of Ibrutinib (orange, PDB code: 5P9J) and Zanubrutinib (green, PDB code: 6J6M) covalently bound to BTK Cys481.

**Figure 3:**
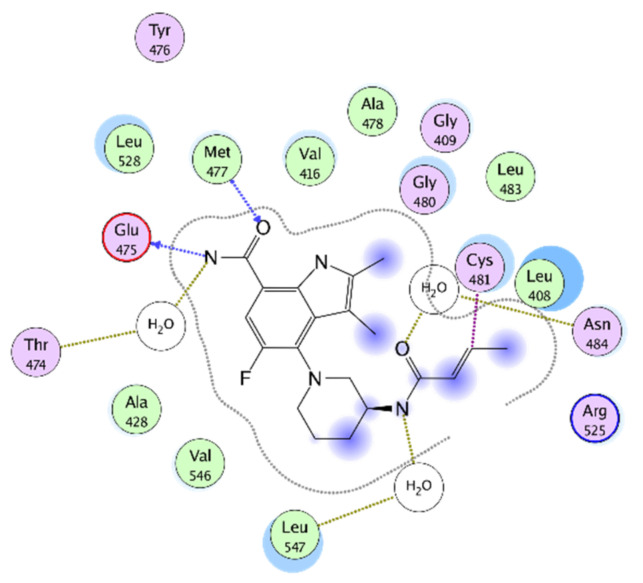
Ligplot of the BTK-Branebrutinib crystallographic complex (PDB code: 6081).

All are characterized by a reactive Michael acceptor group, which is responsible for a major part of the undesired side effects such as allergic reactions, fever, lymphadenopathy, edema and albuminuria [28]. For example, the crystal structures of the approved irreversible BTKIs, Ibrutinib and Zanubrutinib, bound to the BTK enzyme and their covalent interaction with Cys481 (Figure 2). The interactions between BTK and the recent irreversible BTKI Branebrutinib are reported in Figure 3.

Evobrutinib is under clinical investigation for autoimmune disorders and, when orally administered, it showed good efficacy in mouse models of RA and SLE, as demonstrated by a reduction of disease severity and histological damage [29,30].

Spebrutinib shares a common diphenylaminopyrimidine (DPPY) pharmacophore with several tyrosine kinase inhibitors, such as Alk [31], focal adhesion kinase (FAK) [32], and EGFR inhibitors [33,34]. It is under study in combination with FAK inhibitors to treat solid tumors (particularly esophageal cancer) [35].

Remibrutinib exhibits a good kinase selectivity due to binding to a BTK inactive conformation, which demonstrates potent in vivo activity with an EC_90_ of 1.6 mg/kg and dose-dependent efficacy in rat collagen-induced arthritis. It is currently being tested in phase II clinical studies for chronic spontaneous urticaria and Sjögren syndrome [36,37].

Tolebrutinib is now under phase III clinical studies for MS treatment [38,39]. Olmutibib is characterized by a thieno-pyrimidine scaffold; it is under phase II clinical investigations for arthritis treatment [13]. Interestingly, it also inhibits EGFR and for this reason now it is in clinical trials for non-small cell lung cancer (NSCLC) that target EGFR [40].

Branebrutinib is a highly selective covalent, irreversible BTK inhibitor (IC_50_ = 0.1 nM) with rapid target inactivation in vivo. In a low human dose in phase I randomized, placebo-controlled, multiple-ascending-dose studies in healthy participants, it is well tolerated at doses of ≥3 mg orally administered once a day. These data support the continued clinical investigations of this novel agent [41,42]. 

TAK-020 is an orally available irreversible inhibitor with sufficient selectivity over other similar kinases (such as Src and Tec) [43]. Additional in vitro and in vivo assays revealed good pharmacodynamic (PD) and efficacy properties, showing that TAK-020 could be efficacious at a very low human dose. Now, a phase I, randomized, double blind, placebo-controlled, single-dose and multiple-rising-dose study is ongoing [44].

Elsubrutinib inhibits BTK with less potency compared to other compounds [45], but it is able to block catalytic domains in a time-dependent tuneable manner, representing the starting point of new tuneable covalent inhibitors. In addition, Elsubrutinib demonstrated efficacy in pre-clinical models of RA and SLE [46]. This specific action is characteristic of reversible covalent inhibitors, among which Rilzabrutinib is the most relevant.

As previously reported, efforts have been made to discover compounds with reduced side-effects but a high potency, representing the starting point of covalent inhibitors with tuneable residences over time. The previously cited Rilzabrutinib, endowed with a pyrazolo-pyrimidine scaffold, forms a covalent bond with Cys481, but at the same time its inhibition is temporary [4,47,48]. It is under study for its effects on RA, pemphigus vulgaris, and immune thrombocytopenia [7,14].

**Table 2:** Name, chemical structure, IC_50_ values of BTK and clinical phases of irreversible BTKIs under clinical and pre-clinical investigation.

Name	Company	Chemical Structure	IC_50_ or pIC_50_ Values on BTK	Clinical Phases	Ref
Evobrutinib	Merk	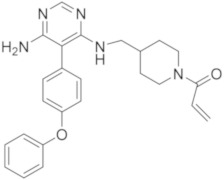	37.9 μM	Phase III	[29,30]
Spebrutinib	Avila Therapeutics/ Celegene	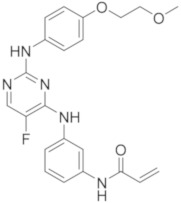	0.5 μM	Pre-clinical studies	[35]
Remibrutinib	Novartis	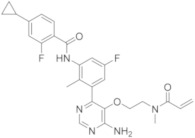	1.3 nM	Phase II	[36,37]
Tolebrutinib	Sanofi/Principia Biopharma	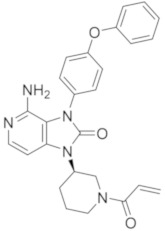	0.4–0.7 nM	Phase III	[38,39]
Olmutinib	Hamni Pharmaceuticals	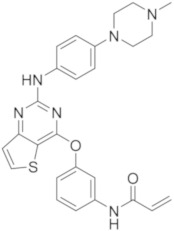	1.0 nM	Phase II	[13,40]
Branebrutinib	Bristol-Myers Squibb	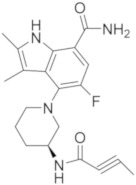	0.1 nM	Phase I	[41,42]
TAK-020	Takeda	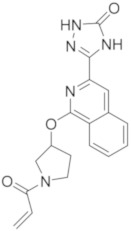	pIC_50_ > 8.7	Phase I	[43,44]
Elsubrutinib	AbbVie	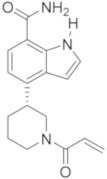	0.18 μM	Phase II	[45,46]
Rilzabrutinib	Sanofi	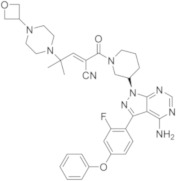	3.1 nM	Phase III	[4,7,14,47,48]

### 4.2. Reversible BTKIs

From a chemical point of view, reversible inhibitors present different chemical scaffolds, such as pyrimidines, 2,4-diaminopyrimidines, and 1,3,5-triazines; as well as condensed structures as pyrazolo-pyrimidines, pyrazolo-pyridines, pyrrolo-pyrimidines, pyrrolo-triazines, imidazo-pyrazines, imidazo-pyrimidines, imidazo-quinoxalines; and purines, such as Vecabrutinib, Fenebrutinib, RN-486, and GNE-431 (Table 3) which are under clinical investigation for haematological disorders and autoimmune diseases [7]. Unfortunately, no reversible BTKIs are currently in the market.

GNE-431 (Table 3), endowed with different heterocyclic moieties, is a very promising molecule, being able to inhibit C481R, T474I, and T474Ms mutants. It represents the first example of a “pan-BTK” inhibitor [49,50].

RN-486 (Table 3) [49,50] is under preclinical investigation, in particular for RA; it presents IC_50_ values of 4nM compared to BTK, as well as some activity compared to Slk and Tec (with IC_50_ values of 43 nM and 64 nM, respectively) [4,51,52].

Bristol-Myers Squibb (USA) researchers in the past were deeply involved in the search of a new reversible BTKI and reported a compound of BMS-935177 and BMS-986142 (Table 3). Both bear a carbazole core which is differently substituted, similar to Branebrutinib and Elesebrutinib. BMS-986142 in particular is an interesting new lead compound that is able to block BTK in reversible manner, with an IC_50_ value of 0.50 nM; in addition, it shows significant improvements in potency and selectivity compared to BMS-935177, and it is currently in a phase II clinical study for the treatment of RA and Sjögren Syndrome [53,54,55,56,57].

CGI-1746 (Table 3) is characterized by a pyrazine core [58]; it is the first reversible BTKI reported to bind to a specific inactive non-phosphorylated BTK conformation, resulting in an excellent kinase selectivity. CGI-1746 shows a good BTK inhibition (IC_50_ = 1.9 nM), inhibiting both auto- and trans-phosphorylation steps necessary for enzyme activation. Using CGI-1746, authors demonstrated that BTK regulates inflammatory arthritis by two distinct mechanisms, by blocking B-cell receptor–dependent B-cell proliferation, and by abolishing TNFα, IL-1β and IL-6 production. These results clarify the function of BTK in both B-cell or myeloid cell–driven disease processes and provide a rationale for targeting BTK in RA.

Genentech and Gilead researchers (USA) in 2008 patented a lot of BTKIs under the name GCD-0834 (Table 3), which retained the potency and selectivity of CGI-1746 with improved PK in preclinical animal models [25,59]. In 2017, they demonstrated that more rigid molecules, bearing a tetrahydrobenzothiophene scaffold, characterized by a less exposed N−H donor and a reduced number of rotatable bonds, could potentially improve potency and permeability. With this purpose, they synthesized G-744, (Table 3) which shows some similarity with previous derivatives of RN-486, but is characterized by a tetrahydropyridinone ring fused with thienocyclopentane-one. G-744 showed excellent potency on BTKs (an IC_50_ value of 0.002 μM), as well as good selectivity and favourable PK properties. In addition, it demonstrated similar efficacy to dexamethasone in a rat collagen-induced arthritis (CIA) model at a 25 mg/kg b.i.d. dose, confirming that it is possible to use as therapeutic agents in autoimmune diseases such as arthritis [60,61].

The same authors also reported compound G-278 (Table 3), in which the thiophene ring of G-274 is substituted by a pyrrole one. It inhibits BTK with an IC_50_ value of 4 nM, showing good kinase selectivity and an acceptable pre-clinical PK profile. Unfortunately, in rat and dog tolerability studies, G-278 induced dose-limiting toxicities with unacceptably low safety margins in both species. In Sprague−Dawley rats, an unusual pancreatic toxicity was also observed, whereas in dogs, liver toxicity was reported; consequently, this compound and its related analogues have been abandoned [62].

To overcome the toxicity problem, the authors modified the structure of G-278 on the basis of deeply crystallographic and molecular modelling studies. They focused their attention on the piperazine tail and were able to increase van der Waals contacts with the edge of the protein. In a subsequent compound, GDC-0853 (Table 3), they introduced a small alkyl substituent (methyl) on piperazino moiety, maintaining the same tricyclic scaffold of the previous compounds G-744 and G-278, but changing the central core of the molecule, inserting a pyridine nucleus instead of a simple benzene ring. 

**Figure 4:**
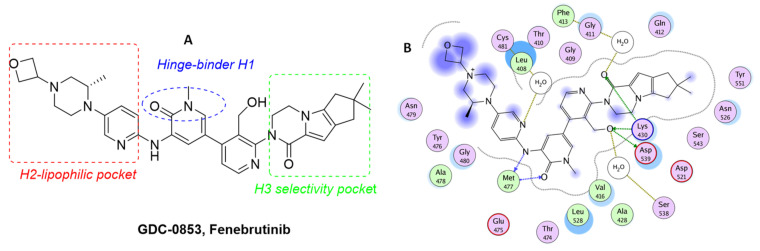
(**A**) Schematic representation of interaction of BTK and Fenebrutinib; (**B**) Ligplot of BTK–Fenebrutinib crystallographic complex (PDB code: 5VFI).

In the schematic representation of the different roles of the substituents of GDC-0853, a BTK interaction is reported (Figure 4). The pyridinone core interacts with the hinge region, whereas a piperazino tail and tricyclic nucleus have a specific role in the interaction with H2 and H3 pockets, respectively. GDC-0853 potently inhibits BTK (IC_50_ = 0.91 nM), suppresses the B-cell- and myeloid cell-mediated components of disease, and demonstrates dose-dependent activity in an in vivo rat model of inflammatory arthritis. In addition, different to previous derivatives, it demonstrates highly favourable safety, PK, and pharmacodynamic (PD) profiles. For these reasons, GDC-0853, under the name Fenebrutinib, is in phase III studies in patients with RA (NCT02983227, NCT02833350), relapsing multiple sclerosis (RMS) (NCT04586023, NCT04586010), primary progressive MS (NCT04544449), severe active SLE (NCT02908100), and chronic spontaneous urticaria (NCT03693625, NCT03137069) [63].

**Table 3:** Name, chemical structure of some reversible BTKIs under clinical or pre-clinical investigations.

Name	Company	Chemical Structure	Clinical Phases	Ref
Vecabrutinib	SNSS	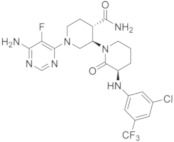	Phase II	[13]
GNE-431	Genentech	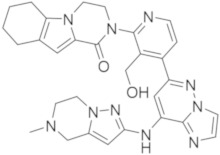	Pre-clinical investigation	[49,50]
RN-486	Roche	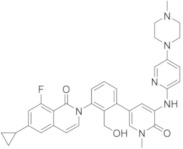	Pre-clinical investigation	[4]
BMS-935177	Bristol Meyers Squibb	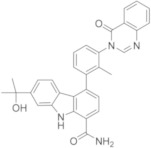	Phase II	[53,54,55,56,57]
BMS-986142	Bristol Meyers Squibb	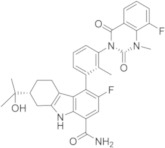	Phase II	[53,54,55,56,57]
CGI-1746	CGI/ Genentech	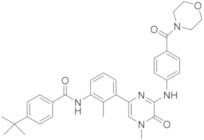	Pre-clinical investigation	[58]
GDC-0834	Gilead/ Genetech	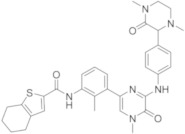	Pre-clinical investigation	[25,59]
G-744	Gilead/ Genetech	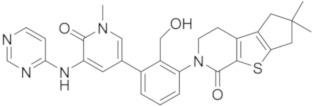	Pre-clinical investigation	[60,61]
G-278	Gilead/ Genetech	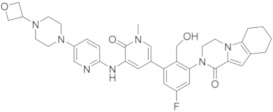	Pre-clinical investigation, now abandoned	[62]
Fenebrutinib	Genetech	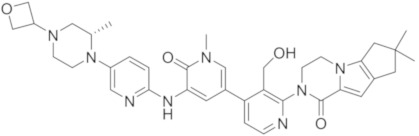	Phase III	[63]

## 5. Recent Advances in BTKIs 

In the past ten years, different chemical scaffolds have been deeply investigated and patented as BTKIs [4,13]. In particular, Feng in 2019 and, more recently, Liu and Zhang [13,15,23] reported complete reviews in which they summarized the most interesting compounds obtained by different companies and academic researchers that were the most useful in cancer therapy as well as in inflammatory and autoimmune diseases. Pyrimidines, 2,4-diaminopyrimidines, 1,3,5-triazines, and condensed structures such as pyrazolo-pyrimidines, pyrazolo-pyridines, pyrrolo-pyrimidines, pyrrolo-triazines, imidazo-pyrazines, imidazo-pyrimidines, imidazo-quinoxalines, and purines gave the best results. The same authors recently published a review focused on BTKIs that were launched in the market and were under clinical evaluation [7]. While there is much data on the role of BTK in malignancies, BTKIs in clinical or in preclinical–clinical investigations have been very recently reported [13,15,23]. A systematic list of compounds in the early phases of study is not yet available. For these reasons, we thought it would be more useful to conduct a systematic report on the main chemical classes investigated in the last five years. In this review, we summarized the recently developed (ir)reversible BTKIs that have appeared in the literature in the last five years (from 2017 to date), covering recent advances in the field of medicinal chemistry**.** Data reported here are collected from different databases (Scifinder, Web of Science, Scopus, Google Scholar, and Pubmed) using “BTK” and “BTK inhibitors” as keywords. Reported compounds are classified by their chemical class, as evidenced in Table 4.

### 5.1. Diphenylaminopyrimidines (DPPYs)

As previously reported, the diphenylaminopyrimidine core has been deeply investigated as a chemical scaffold that is useful in the synthesis of most kinase inhibitors [31,32,33,34]. Currently, Spebrutinib is the most promising compound of these analogues.

**Figure 5:**
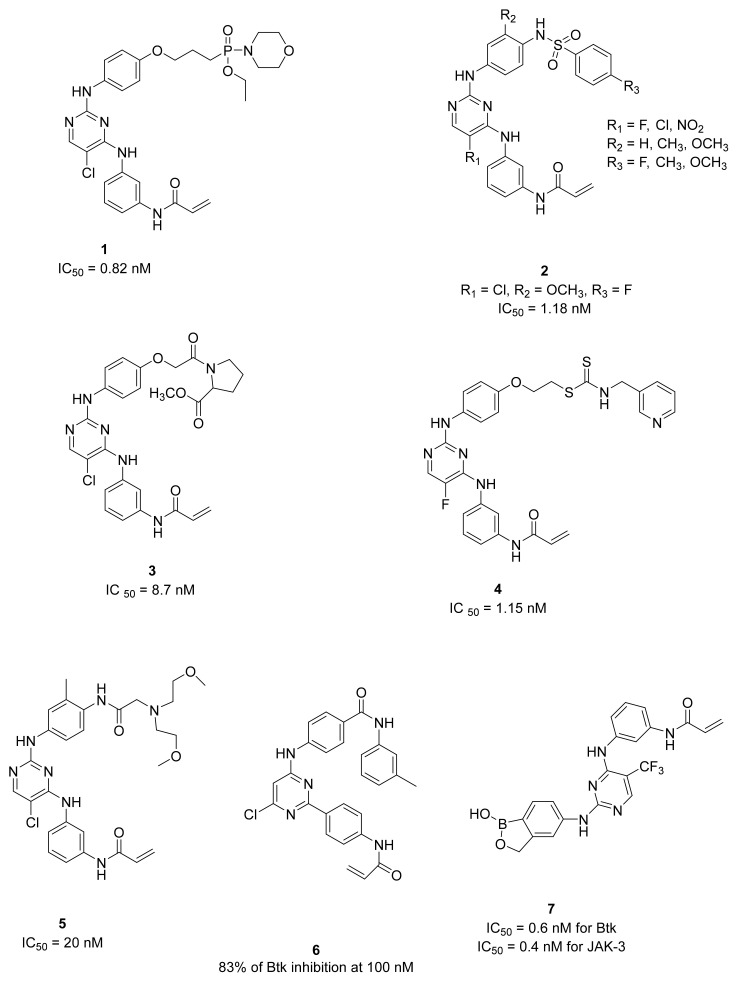
DPPYs reported as BTKIs.

The phosphoryl group has well-known biological functionality and, consequently, it is often used to improve the aqueous solubility and biological activity of anticancer alkylating agents that require in vivo activation [64,65]. In 2017, Ge et al., reported a family of phosphoryl-substituted diphenylpyrimidine derivatives (Pho-DPPYs). The newly introduced phosphoryl group seems to form an additional hydrogen bond, showing that it could ameliorate enzyme interaction, as suggested by the molecular modelling simulation. The most active reported derivative (compound **1**, Figure 5) inhibits BTKs at low nanomolar concentrations (IC_50_ = 0.82 nM), suppress B-cell leukemia proliferations (Ramos and Raji cell lines) at concentrations of 3.17 mM and 6.69 mM, respectively, and blocks cell apoptosis, representing a promising BTK inhibitor for the treatment of B-cell lymphoblastic leukemia [66,67].

The same research group synthesized a new series of DPPYs possessing a functional sulfonamide in the C-2 aniline moiety of the pyrimidine template (SFA-DPPYs). This substituent presents itself in many active compounds such as antibacterial, anti-carbonic anhydrase, diuretic, hypoglycemic, and anticancer compounds [68]. Some of these molecules (Figure 5) displayed high potency against the BTK, with IC_50_ values in the low nanomolar range. In particular, compound **2** inhibits B-lymphoma cell lines with a proliferation stronger than Spebrutinib. In addition, the inhibitory potency towards the normal PBMC cells showed that compound **2** possesses low cell cytotoxicity. All these explorations indicated that SFA-DPPYs could serve as a valuable inhibitor for B-cell lymphoblastic leukemia treatment [69,70]. 

Wang et al., reported a new family of DPPYs bearing an amino acid substituent. Among them, compound **3** (Figure 5), which features an L-proline substituent, resulted in the most active compound, with an IC_50_ of 8.7 nM on BTK enzymes and activity against B-cell lymphoma cell lines, similar to Ibrutinib. Moreover, compound **3** exhibited low cytotoxic activity against normal PBMC cells. In addition, Western blot analyses and flow cytometry analyses also showed its effectiveness in interfering with B-cell lymphoma cell growth. Additional molecular studies evidenced that compound **3** formed additional strong hydrogen bonds with BTKs, providing a new insight into improving the activity of amino acid-substituted DPPYs [71]. 

In 2019, Zhai et al., obtained good results with compound **4** (IC_50_ = 1.15 ± 0.19 nM, Figure 5), which resulted more activity against BTKs than the reference compounds Ibrutinib and Spebrutinib. Interestingly, authors added dithiocarbamate moieties, as in molecules previously reported as FAK inhibitors [72], to the diphenylaminopyrimidine core, which is typical of Spebrutinib. Compound **4** showed enhanced anti-proliferative activity against B-lymphoma Ramos and Raji cell lines with good selectivity, as well as efficiently blocking BTK downstream pathways and stimulating cancer cell apoptosis. In addition, in an in vivo xenograft model, it demonstrated significant efficacy and broad safety [73].

At the same time, other researchers reported a new small library of DPPYs bearing various substituted anilines at the C-2 position of the pyrimidine core as irreversible BTKIs. Most of them exhibited a similar potency to the reference compounds (Spebrutinib and Ibrutinib) in both kinase-based and cell-based assays. The most significant compound was **5** (Figure 5), characterized by a bis(2-methoxyethyl)amino)acetamido moiety on the anilino group, which inhibits the BTK enzyme with an IC_50_ value of 20 nM, as well as blocking Ramos and Raji cell proliferation with IC_50_ values of 10.5 μM and 19.1 μM, respectively, and causing the apoptosis of Ramos cells at the G0/G1 phase unlike the reference compounds which arrested cell cycles at the late phase [74]. 

As previously reported for compounds **1**–**3**, a presence of the chlorine atom on the pyrimidine nucleus seems to be fundamental for biological activity; following this consideration, Li et al., prepared a small library among which the most active compound, compound **6** (Figure 5), which, unlike previous derivatives, bears acrylamido moiety on the para position of the phenylamino ring. It displayed the best inhibitory activity of BTKs (with an inhibition rate of 82.76% at 100 nM) and excellent antiproliferation activity on three B-cell leukemia lines (IC_50_ = 3.66 μM, 6.98 μM, and 5.39 μM against HL60, Raji, and Ramos, respectively). In addition, it blocked the Ramos cells at the G0/G1 phase and further investigations on the intracellular mechanism of action demonstrated that compound **6** could inhibit BTK phosphorylation and its downstream substrate phospholipase γ2 (PLCγ2) [75].

More recently, Ren et al., created a new chemical entity, combining a benzoxaborole scaffold with a pyrimidine pharmacophore. Specifically, they introduced benzoxaborole into the 2- or 4-position of pyrimidine, obtaining a focused library (31 compounds), with dual activities on BTKs and JAK3 (other attractive target for hematologic, malignant, and autoimmune diseases). Through a structural optimization approach, they obtained some compounds which were more active in enzyme and cell-based assays (IC_50_ < 2 nM against both BTKs and JAK3). Particularly, the most promising compound (**7**, Figure 5) was more active than Ibrutinib and in metabolic stability experiments on human liver microsomes, and it showed good stability. In addition, it exerted better effects in haematological and immune diseases through synergy. The further pre-clinical evaluations of compound **7** is underway. These explorations could offer new clues to discover the benzoxaborole fragment and the pyrimidine scaffold as more effective BTK and JAK3 dual inhibitors [76].

All previous compounds **1**–**7** are irreversible inhibitors.

### 5.2. Other Pyrimidine and Pyridine Derivatives

**Figure 6:**
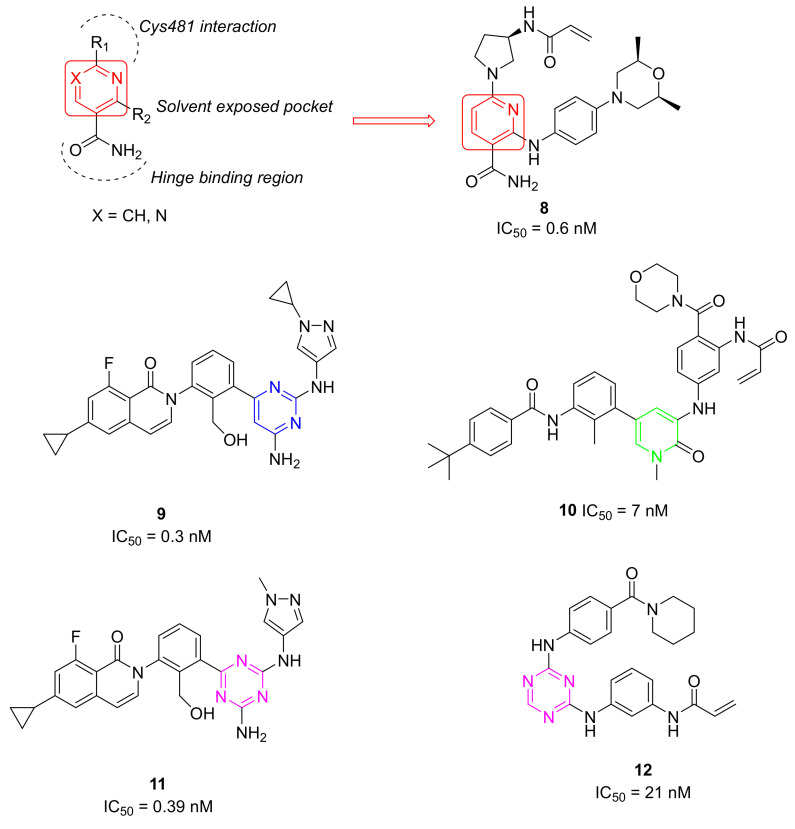
Pyrimidine, pyridine, pyridinone, and 1,3,5 triazine derivatives.

When researchers from the EMD Serono Research and Development Institute (Billerica, MA, USA), Constellation Pharmaceuticals (USA), and F. Hoffmann-La Roche (Switzerland) discovered Evobrutinib in 2018, they reported a series of pyrimidine and pyridine carboxamides as BTK irreversible inhibitors, obtained using X-ray guided structure-based design (SBD). One goal of this work was to discover a suitable replacement for the aminopyrimidine hinge binding moiety present in Evobrutinib. They obtained a new series of pyridine and pyrimidine compounds, bearing a carboxamide group and different substituents in two other positions necessary for binding with the hinge region (Figure 6). These new BTKIs displayed excellent biochemical and cellular potency, superior kinase selectivity, and improved drug-like properties compared to Ibrutinib. Specifically, pyridine compounds showed a better pharmacological profile when compared with pyrimidine ones. The resulting pyridine carboxamides were potent and selective BTKIs with excellent enzymatic and cellular inhibitory activity, similar to Ibrutinib. Compound **8** (Figure 6) showed excellent biochemical, cellular, and whole blood potency (IC_50_ = 0.6 nM against BTKs). In addition, it was relatively selective in a 270-member kinase panel, only inhibiting 9/270 kinases at 1 mM concentration. The intrinsic clearance of compound **8** was dramatically improved in all tested species when compared to that of Ibrutinib. In addition to these encouraging results, compound **8** showed no significant CYP induction and its hERG inhibition (responsible for QT prolongation and cardiotoxicity) was better than that of Ibrutinib. Unfortunately, compound **8** did not attain sufficient oral bioavailability in a low-dose mouse PK study to be considered as a lead compound. Consequently, further optimization of these analogues to improve the PK profile is necessary [77].

Researchers at Carna Biosciences (Japan) obtained a series of novel pyrimidine analogues as potent, highly selective, non-covalent BTKIs by the isosteric substitution of the triazine nucleus with a pyrimidine one. These compounds showed a strict similarity with the previous compound RN-486, bearing the same substituted isoquinoline moiety. Derivative **9** (Figure 6) demonstrated a high affinity for BTKs (IC_50_ = 0.3 nM) and an excellent kinase selectivity, probably due to the binding of the compound to the BTK inactivated conformation. It strongly inhibited BTK autophosphorylation in cells and showed a good oral bioavailability in mice. Unfortunately, compound **9** inhibits the hERG channel; consequently, further optimization to eliminate the hERG issue is necessary [78].

### 5.3. Pyridinone Derivatives

Using structure-based drug design technology, Liang et al., discovered an irreversible, highly selective BTK inhibitor, compound **10** (Figure 6). It is characterized by a pyridinone nucleus that is able to block BTKs at a nanomolar concentration (IC_50_ = 7 nM) and is endowed with high selectivity profile in KINOMEscans among 468 kinases/mutants at 1 mM concentrations. Specifically, compound **10** completely abolished the activities of BMX, JAK3, and EGFR, as well as potently inhibiting BTK Y223 auto-phosphorylation, arresting the cell cycle in the G0/G1 phase, and inducing apoptosis in U2932 and Pfeiffer cells. To elucidate the binding mode of compound **10** with BTKs, a high-resolution crystal structure (1.585 Å) of the BTK domain in a complex with compound **10** was obtained. These studies revealed that compound **10** binds BTK in a DFG-in/C-helix-out inactive conformation, confirming its irreversible binding mode. Additionally, compound **10** forms some additional hydrogen bonds with enzymes, two with the main chain amide and the carbonyl of Met477, and another with the Lys430 side-chain [79,80].

### 5.4. 1,3,5-Triazine Derivatives

1,3,5-triazines represent an interesting scaffold for different kinase inhibitors, including BTK and FAK inhibitors [32].

Kawahata et al., overcame the cardiotoxicity problem that was previously reported for derivatives of compound **9** by using synthesized novel and potent triazine-based inhibitors using a scaffold-hopping approach and detailed SAR studies. They obtained a highly selective BTKI, compound **11** (Figure 6), in which the pyrimidine nucleus of compound **9** was substituted with a triazine one. Compound **11** inhibits the inactive form of BTKs with an IC_50_ value of 0.39 nM; it is also characterized by a significant kinase-selectivity profile. In addition, it exhibited strong inhibitory potency against the BCR activation in B-cells and showed excellent PK profiles in multiple species. In a mouse CIA model, compound **11** significantly reduced paw swelling and joint inflammation in a dose-dependent manner. On the basis of the in vitro potencies, PK profiles, and in vivo efficacies, compound **11** was advanced into pre-clinical development studies [81,82].

Recently, Teng et al., synthesized many new 1,3,5-triazine derivatives and evaluated their biological activities on BTKs. Biological assays, a molecular docking study, and ADME property predictions were made, and a highly potent selective and covalent BTKI has been identified (compound **12**, Figure 6). This new lead compound showed an IC_50_ value of 21 nM against BTK, as well as excellent activity on Raji cells and Ramos cells (IC_50_ values of 5.14 nM and 6.14 nM, respectively). In addition, it potently inhibited BTK Y223 auto-phosphorylation, arrested the cell cycle in the G2/M phase, and induced apoptosis in Ramos cells. The high selectivity for BTKs and high potency in TMD8 cells by compound **12** suggests a low risk of off-target related adverse effects. Further molecular docking and dynamic simulation-furnished insights into its binding mode with BTK are needed [83].

### 5.5. Pyrazolo-Pyrimidine Derivatives

The pyrazolo-pyrimidine nucleus, present in Ibrutinib and Rilazutinib, as previously reported, represents the most studied scaffold, investigated by academia and pharmaceutical companies alike.

**Figure 7:**
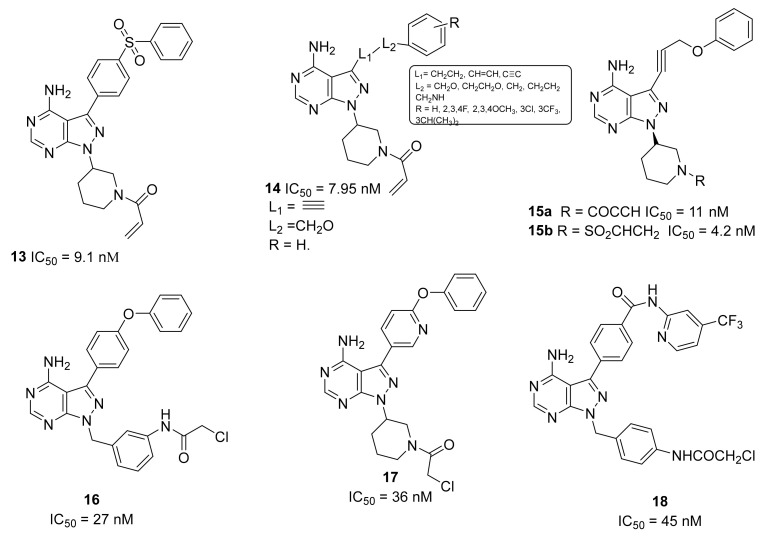
Pyrazolo-pyrimidine derivatives.

Park et al., reported a novel series of pyrazolo-pyrimidine derivatives with a sulfone group inserted in the phenyl moiety on C-3 of the pyrazole nucleus. The most active compound, compound **13** (Figure 7), exhibited the same inhibitory activity against BTK as Ibrutinib (IC_50_ = 9.1 nM) and displayed a potent antiproliferative activity in the TMD8 cell-based assay. Authors also performed a docking study to investigate the binding mode of compound **13** compared with Ibrutinib; these investigations evidenced that, as expected, the pyrazolo[3,4-*d*]pyrimidine scaffold of compound **13** made two hydrogen bonds with Met477 and Glu475 in the hinge region, whereas the diphenyl sulfone group extended towards a back pocket and formed a hydrophobic interaction with Met449, Leu460, Ile472, and Phe540. Additionally, sulfone moiety maked a hydrogen bond with Lys430. Authors also suggested that the acrylate moiety made a covalent bond with Cys481, forming a hydrogen bond with a backbone amide group of Cys481. Further PK and in vivo animal model studies are projected, but not reported [84].

Zheng et al., reported the synthesis and biological evaluation of a small library of 3-substituted 4-amino-1*H*-pyrazolo[3,4-*d*]pyrimidine derivatives bearing C-3 alkyl side chains as novel potent BTKIs. In this research, the backbone and the groups necessary for irreversible activity were retained, whereas in the C-3 position the phenyl ring that is typical of Ibrutinib was replaced by three carbon groups, propanyl, allyl, and propynyl, and four atoms, including O, C, N, and S as the connection of the C-3 with the final phenyl ring in order to maintain the bioactive conformation of the distal phenyl group and to decrease some of the hydrophobic characteristics of Ibrutinib. Most of the synthesized compounds displayed good inhibitory activities against both BTK and B-cell lymphoblastic leukemia lines in vitro. Among them, compound **14** (Figure 7) exhibited excellent potency (IC_50_ = 7.95 nM against BTK enzymes, 8.91 μM against Ramos cells, and 1.80 μM against Raji cells), with better hydrophilicity (ClogP = 3.33). These investigations provided new information to discover C-3-substituted pyrazolo-pyrimidine derivatives as novel anticancer agents [85].

Starting from previous compound, compound **14**, the same authors more recently reported a structural optimization study in which they analysed the space near the piperidinyl group on the pyrazole cycle. The molecular docking simulations in fact indicated that when this space is occupied with an appropriate ring or flexible side, an ameliorated BTK inhibition could be obtained. For this purpose, authors synthesized a new series of 1-substituted pyrazolo[3,4-*d*]pyrimidine derivatives as novel potent BTKIs. Among them, compounds **15a** and **15b** (Figure 7) displayed a strong activity with IC_50_ values of 11 nM and 4.2 nM, respectively. In particular, compound **15a** remarkably suppressed the proliferation of DOHH2 and WSU-DLCL2 cells lines, showing stronger inhibitory activity than Ibrutinib. These studies evidenced that this class of pyrazolo[3,4-*d*]pyrimidine derivatives could represent a promising scaffold to obtain new irreversible BTKIs [86].

Ran et al., on the basis of the general structure of BTKIs that have previously been reported (Figure 1) [26,27], designed a new series of compounds in which the backbone and hydrophobic groups of Ibrutinib were retained, whereas the piperidine linker was replaced by a phenyl ring which was attached to the nucleus via a methylene group. Among these compounds, derivative **16** (Figure 7) exhibited potent BTK inhibition (IC_50_ = 27 nM), high selectivity, antiproliferative effects in primary patient tumor cells, and strong apoptosis induction in Jeko-1 and Z138 cells. Specifically, **16** showed antiproliferative activities in MCL cell lines with IC_50_ values lower than 1 μM [87].

In 2020, the same authors synthesized a novel series of 3-(6-phenoxypyridin-3-yl)-4-amine-1*H*-pyrazolo[3,4-*d*]pyrimidines, which are chemically very similar to Ibrutinib and the previous derivative, compound **16**. The most potent compound (**17**, Figure 7) in which they introduced a pyridine nucleus instead of phenyl one in C-3, showed potent BTK inhibition (IC_50_ = 36 nM) and completely inhibited PLCγ2 phosphorylation in Z138 cells at a low micromolar concentration, as well as exerting antiproliferative activities in MCL cell lines (IC_50_ values < 1 μM), and inducing cell apoptosis in Jeko-1 and Z138 cells. In addition, compound **17** seemed to have greater BTK selectivity and a higher stability in human liver microsomes compared to Ibrutinib, confirming its potential and safe use in MCL treatment [88].

Very recently, the same authors reported on compound **18**, which exhibited potent BTK inhibitory activity (IC_50_ = 45 nM) and enhanced antiproliferative activity compared to Ibrutinib against MCL cell lines (IC_50_ values lower than 1 μM). The low micromolar doses of compound **18** inhibited the BCR signalling pathway, and strongly induced apoptosis and autophagy in a dose-dependent manner in Z138 cells. Moreover, compound **18** induced the production of reactive oxygen species (ROS), probably due to its potent antiproliferative activity. Importantly, compound **18** showed greater BTK selectivity than Ibrutinib, indicating a potentially safer treatment of MCL [89].

### 5.6. Tieno-Pyrimidine Derivatives

Moreover, the thieno[3,2-*d*]pyrimidine scaffold has been extensively studied as an effective pharmacophore for BTK inhibition, but its substituted derivatives are rarely reported.

**Figure 8:**
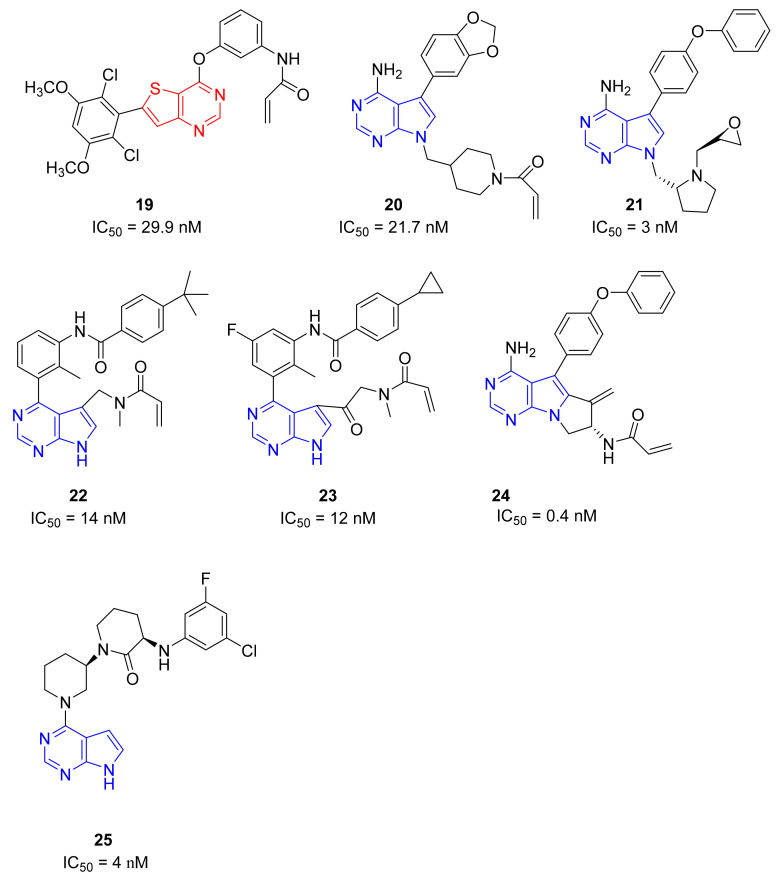
Thieno and pyrrolo-pyrimidine derivatives.

Zhang et al., started from molecules with a thieno[3,2-*d*]pyrimidine core active as an antiproliferative agent [90]. They synthesized new compounds bearing different aryl substituents on the thienyl nucleus. These derivatives exhibited inhibitory activities against BTK in vitro and in particular compound **19** (Figure 8) showed an IC_50_ value of 29.9 nM against BTKs and excellent immunosuppressive action with low cytotoxicity. In addition, compound **19** displayed considerable selectivity between T-cells and B-cells. Furthermore, the enzymatic inhibition on the panel of more than 20 kinases confirmed that compound **19** is more selective than the reference compound Olmutinib, as it is inactive on EGFR, JAK3, and ITK. These preliminary results suggest that compound **19** could be a novel promising lead compound for further evaluation and that the modification of the 4,6-substituted thieno[3,2-*d*]pyrimidine scaffold could be an interesting drug discovery strategy to obtain new selective BTKIs [91,92].

### 5.7. Pyrrolo[2,3-d]pyrimidine Derivatives

Different 7*H*-pyrrolo[2,3-*d*]pyrimidine derivatives were recently synthesized as BTKIs.

In 2018 He and coll. identified compound **20** (Figure 8) as covalent irreversible BTKIs (IC_50_ = 21.7 nM) with moderate BTK selectivity and low cytotoxicity towards HEK293, LO2 and THP-1 cells. In addition, **20** did not inhibit hERG channel and exhibited potent anti-arthritis activity and similar efficacy to Ibrutinib in reducing paw thickness in CIA mice. Therefore, this pyrrolo-pyrimidine compound could represent a potent, selective, safe and durable BTKI with a potential application in arthritis treatment [93].

Starting from these studies, the same authors synthesized a large library of compounds with very potent action on B-cell lymphomas (Ramos and Jeko-1) and Daudi cell lines with high BTK expression. Compound **21** (Figure 8) showed excellent potency against BTK (IC_50_ = 3.0 nM), good kinase selectivity, and an anti-arthritic effect on CIA models in vivo at 60 mg/kg dose, not causing any bone and cartilage morphology changes. Molecular simulation investigations showed that it could be appropriately embedded into the BTK active pocket with a ‘T’ conformation, in which the phenoxy group would be located at the bottom of the pocket, and the epoxy chain branch and pyrrolidine ring portion would be positioned at the opening of the pocket. In addition, the terminal phenyl group would be twisted out of the connecting phenyl ether plane to enter a hydrophobic pocket mainly formed by the N-lobe residues and formed π-stacking interactions with the Phe540 residues of this pocket. As predicted, the oxygen atom in the epoxy structure of the specific enantiomer in compound **21** could form a hydrogen bond, whereas the nitrogen atom of the pyrrolidine moiety (in a protonated form) could cause another hydrogen bond with the nearby Leu408 residue. In general, authors demonstrated that the 7*H*-pyrrolo[2,3-*d*]pyrimidine-4-amine skeleton also could make hydrogen bond interactions with gatekeeper residues of Glu475 and Met477 [94].

Pulz et al., (Novartis, Basel, Switzerland) reported new potent and selective covalent BTKIs with in vivo activity designed by converting pyrrolo-pyrimidine-based selective reversible inhibitors into selective irreversible BTKIs. They combined the highly specific binding mode of reversible inhibitors with the potency of irreversible BTKIs by attaching an electrophilic warhead to reach Cys481. Authors focused the attention on the linker between the pyrrolo-pyrimidine nucleus and the acrylamide warhead to verify if the linker modification could increase the metabolic stability and also improve the potency by providing a more optimal orientation of the warhead toward Cys481. The most active derivatives were compounds **22** and **23** (Figure 8) that showed good BTK inhibition (IC_50_ values of 14 and 12 nM, respectively) and an excellent kinase selectivity, including several Cys-containing kinases. Unfortunately, due to the very high clearance of these compounds, the company decided to discard this scaffold [95].

By merging the pyrazolo[3,4-*d*]pyrimidine nucleus into a tricyclic skeleton, Xue et al., synthesized two types of compounds characterized by a more rigid structure. Among them, isomer **24** (Figure 8) was identified as the most potent, showing an IC_50_ value of 0.4 nM against BTK and 16 nM against BTK-dependent TMD8 cells. Compared to Ibrutinib, isomer **24** was slightly more selective and in TMD8 cells it significantly arrested cell cycle progression at the G1 phase in a dose-dependent manner. In addition, in a TMD8 cell derived animal xenograft model, isomer **24** reduced tumor volume more efficiently than Ibrutinib. Isomer **24** likely covalently binds to Cys481, as confirmed by molecular modelling studies performed by the authors. It overlapped well with Ibrutinib, including the bicyclic skeleton as well as the hydrophobic moiety. In particular, the SH group of Cys481 rotated about 68° to adapt to the newly formed covalent bond between the acrylamido moieties of isomer **24**. Critical hydrogen bonds with hinge residues at Met477, Glu475, and Thr474 were also observed, whereas additional π−π interactions between the terminal phenyl group and Phe540 were reported. All these results suggest that isomer **24** as a new BTKI worthy of further profiling [96,97].

More recently, Hopkins described the new pyrrolo-pyrimidine compounds as BTKIs. Specifically, the most active compound, compound **25** (Figure 8) is characterized by a piperidino moiety in the 4th position of pyrrolo-pyrimidine ring, similarly to Vecabrutinib. Isomer **25** showed a good inhibition of BTK (IC_50_ = 0.004 μM), interesting biological and ADME properties, in vivo metabolic stability, and good oral exposure in rodents. For all these reasons, isomer **25** could represent a good starting point to obtain new reversible BTKIs [98].

### 5.8. Imidazo-Pyrazine, Imidazo-Pyridine and Imidazo-Pyrazole Derivatives

**Figure 9:**
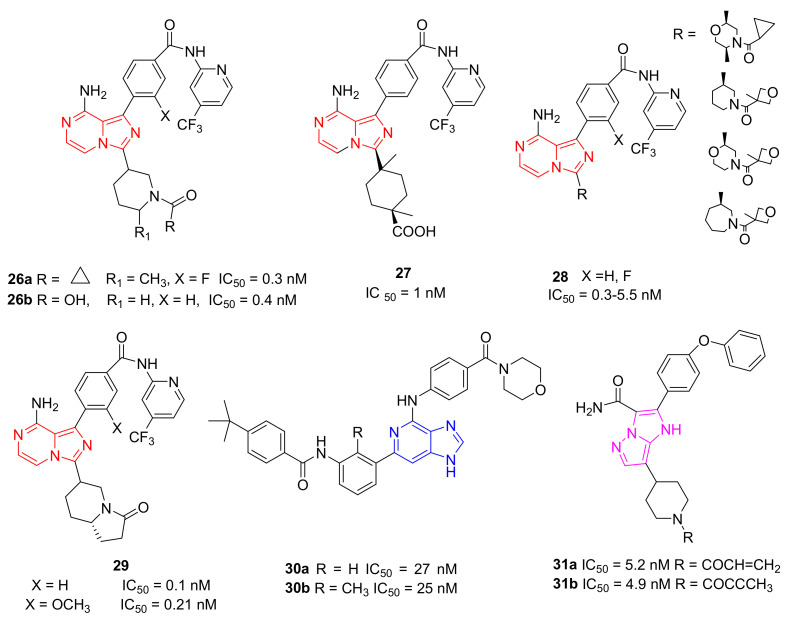
Imidazo-pyrazine, imidazo-pyridine and imidazo-pyrazole derivatives.

Researchers at Merk recently reported the discovery of potent and selective reversible non-covalent BTKIs characterized by an 8-amino-imidazo[1,5-*a*]pyrazine core (compound **26**, Figure 9). These derivatives showed high enzymatic potency (IC_50_ in the low nanomolar range), selectivity, favourable PK properties, andoral efficacy in reducing paw thickness in the CIA rat model. However, compound **26a** resulted in a potent inhibitor of adenosine uptake (AdU) activity, probably responsible for unfavourable cardiovascular effects. An X-ray crystallographic analysis with BTK showed that the piperidine amide moiety occupies the ribose binding pocket of the enzyme; consequently, authors carried out extensive SAR studies replacing the amide with the carboxylic acid moiety. Compound **26b** (Figure 9) resulted in a good BTKI, with superior off-target selectivity and reduced AdU inhibition. Later, to further optimize potency, selectivity parameters, PK parameters, and to reduce the AdU activity, authors reported other reversible BTKIs with good kinase selectivity and acceptable oral bioavailability. Compound **27** (Figure 9) was the most interesting; like the previous compound, compound **26b**, it is characterized by a carboxylic function and inhibits BTKs with IC_50_ value of 1 nM, with reduced side effects [99].

The same authors also reported 8-amino-imidazo[1,5-*a*]pyrazine compounds that differently substituted in position 3. Specifically, guided by SAR studies, they modified the ring size of the groups at the 3-position of the imidazo-pyrazine core, introducing more embedded cycles and a morpholine one, as well as obtaining new agents (compound **28**, Figure 9) endowed with excellent BTK potencies (IC_50_ values in the low nanomolar range), kinase and hERG selectivity, and PK profiles [100].

Pursuing this research, more recently the same company reported more embedded 8-amino-imidazo[1,5-*a*]pyrazines as reversible BTKIs, characterized by bicyclic ring substituents (compound **29**, Figure 9). In this way, the carbonyl group of this bicyclic ring was able to form a hydrogen bond with the C481 backbone amide NH, as demonstrated in the X-ray crystal structure. At the same time, authors observed, for these compounds, an additional interaction with N484. Various bicyclic rings were introduced, showing interesting BTK inhibition in the enzymatic, cellular, and whole blood assays (IC_50_ values were inferior to 1 nM). In addition, new synthesized compounds showed good selectivity against Tec and Src family kinases. These chemical modifications improved potency, as well as the PK profile and off-target selectivity. Two of the most active synthesized compounds, compound **29** (Figure 9), showed good activity in the rat CIA model [101].

Jorda and Krajcovicova designed and prepared five isosteres in which the imidazo[1,2-*a*]pyrazine scaffold of Entospletinib (a well-known Syk inhibitor) was substituted with different bicyclic scaffolds. Imidazo[4,5-*c*]pyridine and its purine derivatives resulted in weaker Syk inhibitors, but simultaneously targeted BTKs more effectively than Entospletinib. Additional experiments showed that the compounds suppressed CBR signalling in Ramos cells, confirming the hypothesis that pan-kinase inhibitors could produce more robust responses in the treatment of B-lymphoid neoplasms [102]. From these investigations, more recently, authors reported an efficient synthetic approach to obtaining trisubstituted imidazo[4,5-*c*]pyridines that can be differently decorated in the N1, C4, and C6 positions. They demonstrated that the imidazo[4,5-*c*]pyridine core exhibited a significantly higher activity against BTKs compared to the imidazo[4,5-*b*]pyridine one, and that imidazo[4,5-*c*]pyridine compounds had a remarkably high tolerance on the C6 position for both hydrophobic and hydrophilic substituents, representing an interesting starting point for the further development of BTKIs that could be useful after Ibrutinib failure. The most active compounds are compounds **30a**,**b** (Figure 9) with IC_50_ values in the nanomolar range (25 and 27 nM, respectively) [103].

More recently, Zhang et al., starting from Zanubrutinib structure, designed and synthesized a small library of imidazo-pyrazole-3-carboxamide derivatives as irreversible BTKIs. The most active compounds were compounds **31a** (IC_50_ = 5.2 nM) and **31b** (IC_50_ = 4.9 nM) (Figure 9) that inhibited BTK auto-phosphorylation, blocked the cell cycle in the G0/G1 phase, induced apoptosis in the TMD8 cells, and suppressed the tumor growth in a TMD8 cell xenograft model. From these biological investigations, the imidazo-pyrazole core emerged as an interesting scaffold to obtain new irreversible BTKIs [104].

### 5.9. Quinoline and Isoquinoline Derivatives

As previously reported for compounds RN-486 and G-744, quinoline and isoquinoline scaffolds represent an interesting nucleus to obtain BTKIs.

**Figure 10:**
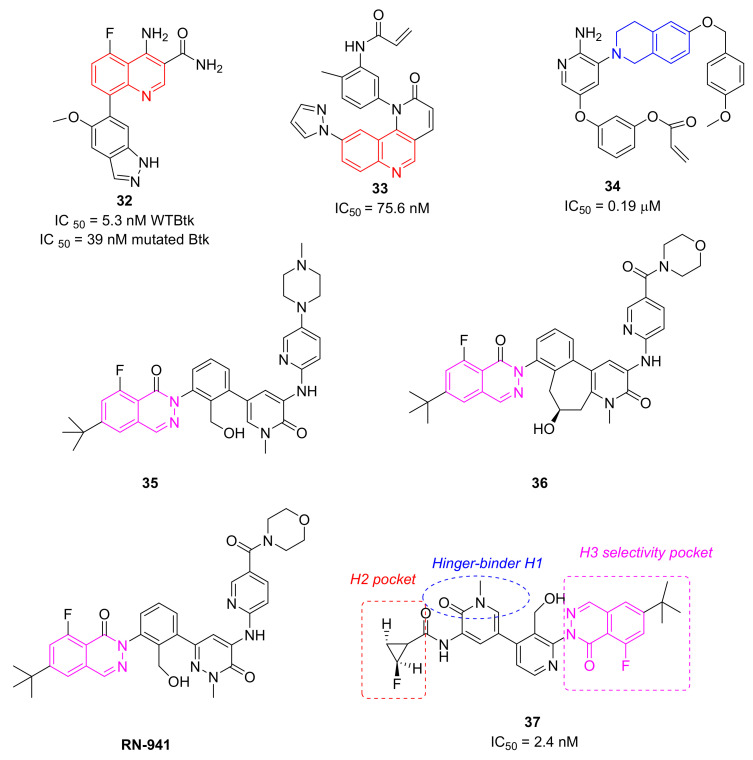
Quinoline, isoquinoline and phthalazine derivatives.

Yao et al., in 2019 applied a structure-hopping strategy, starting from cinnoline compounds, published a new series of 4-amino-quinoline-3-carboxamide derivatives as reversible BTKIs that are useful in the treatment of autoimmune diseases. The most potent compound **32** (Figure 10) possesses a quinoline core linked to an indazole scaffold; it displayed a stronger inhibitory effect on both BTK WT (wild type) (IC_50_ = 5.3 nM) and mutated C481S (IC_50_ = 39 nM) and improved kinase selectivity (particularly against EGFR, TEC, and ITK) compared to Ibrutinib. In addition, a molecular modelling simulation of compound **32** in the active site of BTK suggested that the binding pose was orthogonal to that of Ibrutinib. Finally, in a rodent CIA model, compound **32** efficiently reduced paw swelling without a loss in body weight, and more interestingly, it showed drug-like properties and stability different to previous studied compound such as RN-486 and GDC-0834 [105].

Starting from a quinoline core of the mTOR inhibitor, through a structure-based drug design approach, authors discovered novel irreversible BTKIs characterized by a tricyclic core pharmacophore in which a quinoline ring is fused with a pyridinone one. Interestingly, an additional pyrazole tail was inserted on tricyclic central scaffold. Authors reported and patented a series of compounds with good action and selectivity on the BTK enzyme, where compound **33** (Figure 10) is the most promising. Compound **33** inhibited BTK with an IC_50_ value of 75.6 nM and showed great selectivity over other kinases such as BLK, EGFR, and JAK3. In addition, it potently inhibited BTK Y223 auto-phosphorylation, arrested the cell cycle in the G0/G1 phase, and induced apoptosis in Ramos, MOLM13, and Pfeiffer cells, representing a new interesting pharmacological tool for studying BTK-related pathologies [106,107].

More recently, Lee et al., designed a novel series of BTKIs, endowed with a dihydroisoquinoline scaffold and an electrophilic warhead, which can occupy the allosteric pocket induced by the DFG-out conformation and they are also able to block BTK in its inactivated form. Specifically, derivative **34** (Figure 10) showed good BTK inhibition (IC_50_ values in the low micromolar range), selectivity, and antiproliferative actions on different haematological cell lines. In addition, compound **34** significantly reduced tumor size in a Raji xenograft mouse model, with a 46.8% inhibition compared with the vehicle [108].

### 5.10. Phthalazine Derivatives

Research at Hoffmann-La Roche Inc. has involved the synthesis of different isoquinolin-one derivatives such as RN-486, which are synthesized with similar compounds to minimize the risk of the reactive metabolite formation of previous derivatives, as well as to maximize the benefits, in particular for chronic disease. In 2017, compound **35** (Figure 10), characterized by a phthalazine core, was reported; compound **35** showed a good pharmacokinetic and pharmacodynamic profile, but unfortunately it was found to form glutathione adducts in GSH assays and covalent binding to hepatic microsomal proteins [109].

More recently, the same company synthetized a lot of phtalazine derivatives using the SBD approach. In derivative **36** (Figure 10) the pyridone 6-position is also strategically substituted to suppress the formation of reactive metabolites. Supported by molecular modelling studies, they locked the pyridine system through the formation of a connective seven-membered ring, mimicking the bioactive conformation of similar compounds in BTK active site. In this manner, authors obtained a new cyclic chiral compound, compound **36**, which is endowed with the high potency of previous compounds, but no activity at either glutathione GSH or CYP3A4 (TDI assays), suggesting no formation of reactive metabolites. In addition, the conformation of compound **36** into BTK enzyme was confirmed by an X-ray crystallography study [110].

Simultaneously, a pyridone ring of isoquinoline scaffolds was substituted with pyridazinone one to prevent the formation of reactive metabolites responsible for many adverse drug reactions including idiosyncratic toxicities. In 2017, compound RN-941 (Figure 10) was reported; it showed a good PK and PD profile and displayed a reduced covalent binding to hepatic microsomal proteins (CYP3A4 time dependent inhibition assays, TDI), but unfortunately it was found to form glutathione adducts and covalent binding to hepatic microsomal proteins [109,110].

To develop additional reversible BTKIs free of the toxicity problem, including liver injury, recently the same authors identified other compounds with structural diversity from Fenebrutinib. Since the H3 tricyclic motif and the hydroxymethyl pyridyl ring linker were extensively optimized during the last years, authors explored the solvent exposed H2 region of the previous molecules and in detail they replaced the pyridine ring of Fenebrutinib with a suitably substituted amide. In particular, they hoped that a small cyclopropane carboxamide, which are isosteres of the 2-aminopyridyl group, might replicate the sp^2^ character of the pyridyl π system and that these new amide analogues would benefit from a lower molecular weight, lipophilicity, the lack of a basic amine, and improved aqueous solubility. SAR investigations and its subsequent combination with a (*t*-butyl)-phthalazinone (which is able to interact with the H3 pocket) led to the identification of compound **37** (Figure 10), a potent BTK inhibitor (IC_50_ = 2.4 nM) with excellent physicochemical properties and kinase selectivity. The X-ray cocrystal structure of compound **37** with BTK confirmed that compound **37** formed extensive van der Waals and hydrogen bonding interactions within the active site; as predicted, it formed hydrogen bonds between the pyridone carbonyl and the Met477 amide backbone NH of the hinge region, whereas the cyclopropane ring fits well in the H2 region. While druglike properties were retained and, in some cases, improved, unfortunately a hERG inhibition was observed. In addition, authors evidenced that when a fluorocyclopropyl amide was incorporated, BTK and off-target activity seems to be stereodependent, with the (R,R)-stereoisomer as the most active [111].

### 5.11. Carbazole, Tetrahydrocarbazole, and Indole Derivatives

**Figure 11:**
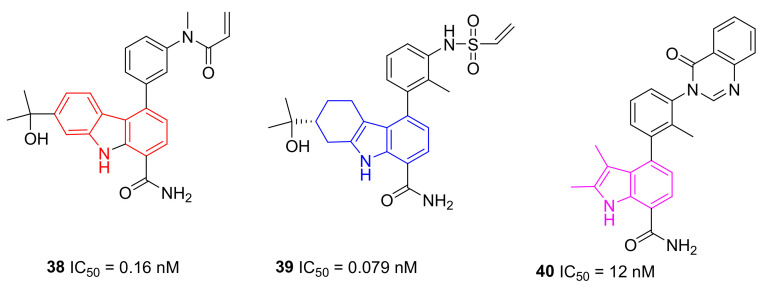
Carbazole, tetrahydrocarbazole and indole derivatives.

Starting from previously reported BMS-935177 and BMS-986142 (Table 3), Bristol researchers incorporated a suitably electrophilic group at C-3′ of the pendent 4-phenyl group of carbazole-1-carboxamide and tetrahydrocarbazole-1-carboxamide cores, transforming these reversible BTKIs into potent, irreversible inhibitors. Specifically, newly synthesized acrylamides in compound **38** and vinyl sulfonamides in compound **39** (Figure 11) displayed sub-nanomolar potency against isolated BTK (IC_50_ values of 0.16 nM and 0.079 nM, respectively) and were also very potent in a cell-based calcium flux assay.

In addition, the removal of one ring from the core of these compounds provided a potent irreversible series of 2,3-dimethylindole-7-carboxamides, which have excellent potency and improved selectivity, with the additional advantages of reduced lipophilicity and molecular weight. The most interesting derivative was **40** (Figure 11) that showed an IC_50_ value against BTK of 12 nM. In summary, new reported derivatives could be starting points for further development [112].

### 5.12. Miscellaneus Compounds

From 2018, many authors reported some (ir)reversible BTKIs with different chemical scaffolds as pyrano-chromenones, benzofuro-pyridines, pyrazoles, and thiazoles.

**Figure 12:**
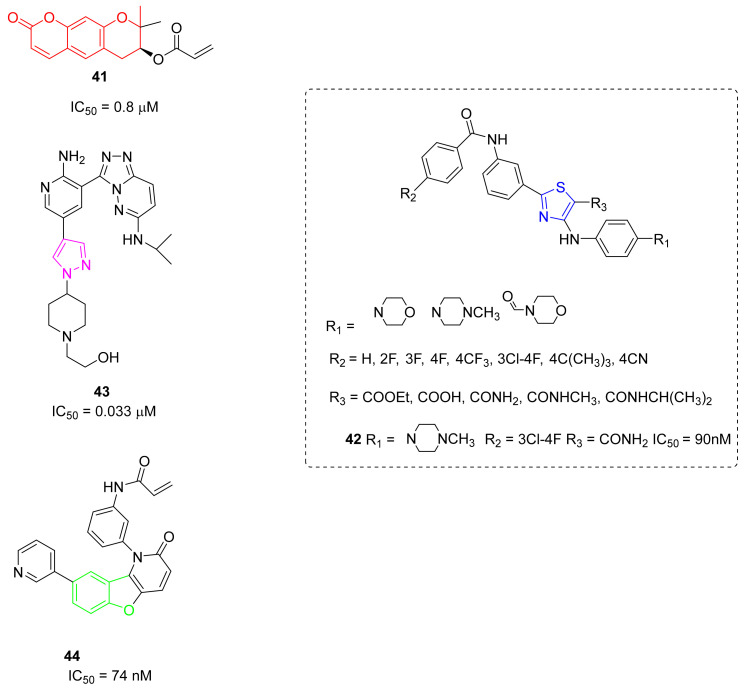
Miscellaneus derivatives.

Cho et al., screened an in-house library of natural products to identify new BTKIs with innovative chemical scaffolds. From these studies, a pyrano-chromenone compound, derived from a natural active component decursin, was found to be active and was selected for further optimization. Consequently, a series of pyrano-chromenone analogues was synthesized through the modification at the C-7 position. Specifically, pyrano-chromenone compounds with an electrophilic warhead showed interesting BTK inhibitory activity, with IC_50_ values in the range of 0.5–0.9 μM, where derivative **41** (Figure 12) was the most promising. Compound **41** demonstrated good selectivity over other associated kinases and decreased the production of proinflammatory cytokines in THP cells. Moreover, compound **41** presented significant in vivo efficacy in a CIA murine model [113].

To discover novel BTKIs with higher efficacy on mutant forms, Guo et al. used a ring-opening strategy and synthesized new a entity bearing a thiazole substituted scaffold able to form an intramolecular hydrogen bond, mimicking, in this manner, a bicyclic structure of Ibrutinib; at the same time, phenyl-linked amide moiety of previous reported CGI-1746 was inserted on the thiazole ring to occupy the H3 pocket of the enzyme. In this manner, they designed and synthesized novel compound **49** diphenylthiazole derivatives, most of which resulted in good BTKIs (IC_50_ values were in the low micromolar range), and antiproliferative agents on B-lymphoma cell lines, and Ramos and Raji cell lines. Unfortunately, the most active compound, compound **42** (IC_50_ = 90 nM) (Figure 12), resulted in less activity than Ibrutinib and CGI-1746 [114].

In 2018, Park et al., reported and patented a new series of aminopyridin-1,2,4-triazolopyridazine derivatives able to block BTK and TMD8 cell lines at low micromolar levels. Specifically, they identified new compounds characterized by an additional substituted pyrazole nucleus linked to an amino-pyridinil moiety (compound **43**, Figure 12). Compound **43** showed an IC_50_ value of 0.033 μM against BTK and a value of 0.8 μM in TMD8 cell lines; further studies on its PK profile and its effects in in vivo animal models are ongoing [115,116].

Other Chinese researchers focused their attention on compounds able to inhibit BTK and PI3Kδ, which both play crucial roles in the progression of leukemia; authors synthesized dual BTK/PI3Kδ inhibitors with the benzofuro[3,2-*b*]pyridine-2(1*H*)-one scaffold as anticancer agents that are more potent comparedto single targeted therapies. Particularly compound **44** (Figure 12) is characterized by a tricyclic benzofuro[3,2-*b*]pyridine core decorated with different aromatics rings. It inhibits, in a irreversible manner, BTK (IC_50_ = 74 nM) and the proliferation of Raji and Ramos cells with IC_50_ values of 2.1 μM and 2.65 μM, respectively. In addition, it showed good selectivity, representing a good lead compound for further optimization as an anti-leukemic drug [117].

## 6. PROTAC Molecules

**Figure 13:**
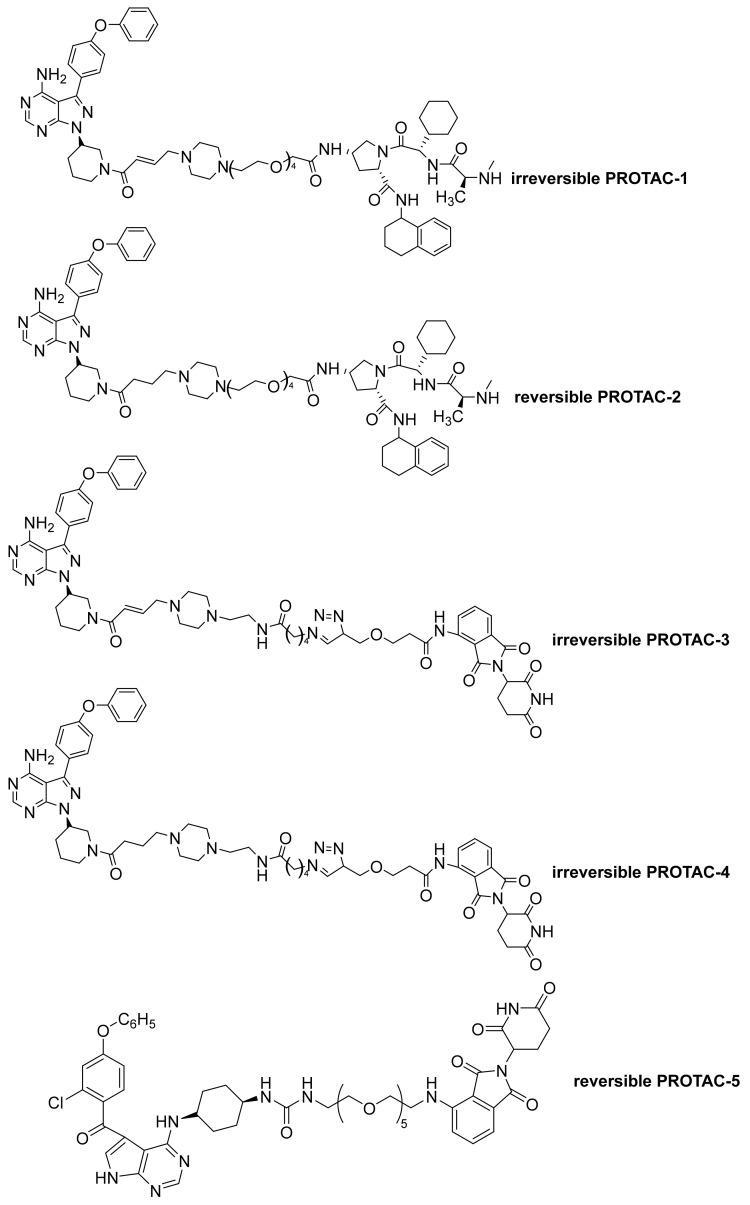
PROTAC derivatives.

In 2019, Tinworth et al., reported the synthesis and biological evaluation of some PROTAC (proteolysis-targeting chimera compounds) derivatives (PROTAC 1–4, Figure 13). In detail, they prepared both irreversible covalent (PROTAC 1 and PROTAC 3) and reversible (PROTAC 2 and PROTAC 4) compounds derived from Ibrutinib. Interestingly, authors evidenced, through proteomics analyses, that irreversible PROTAC molecules did not result in the degradation of covalently bound targets. On the contrary, degradation was observed for reversibly bound targets. This observation highlights a potential caveat for the use of covalent target binders in PROTAC design [11].

More recently, Zhao, to improve the degradation of both WT and C481S mutant BTKs, reported new PROTAC molecules based on the reversible non-covalent BTK inhibitor. New PROTAC derivatives inhibited WT and mutated BTK with high selectivity; particularly, the most active molecules resulted in PROTAC 5 (Figure 13), which strongly causes BTK-WT and BTK-C481S degradation, and the inhibition of BTK-WT and BTK-C481S TMD8 cell proliferation; in addition, it showed moderate membrane permeability and good plasma stability, providing a basis for developing new and potent reversible non-covalent PROTAC-based therapeutic molecules able to treat B-cell lymphomas in particular [12].

## 7. Future Directions

The first approved BTKI, Ibrutinib, has represented an important turning point for the treatment of B-cell malignancies. From 2013, other irreversible BTKIs (Acalabrutinib, Zanubrutinib, Tirabrutinib, and Orelabrutinib) have been launched in the market and represent the most effective drugs in several B-cell malignancies. Unfortunately, the onset of different side-effects and BTK mutations in the active site (Cys481), gatekeeper (Thr474), and SH2 (Thr316) domains could lead to tumor cell proliferation. In fact, all approved BTKIs target Cys481; therefore, simply switching to a another currently approved inhibitor may be ineffective when resistance emerges.

To date, a plethora of reversible BTKIs (Spebrutinib, Evobrutinib, and many others) are under clinical investigation for long-term drug administration for the treatment of autoimmune diseases, especially RA and MS. At the same time, new tuneable BTK irreversible inhibitors such as Rilzabrutinib, or reversible inhibitors able to bind to an inactive non-phosphorylated BTK conformation such as CGI-1746, could represent a promising class of compounds with reduced side effects compared to classical irreversible compounds, such as being able to treat hematological cancers that are Ibrutinib-resistant, as well as chronic pathologies such as autoimmune disorders (i.e., RA, SLE, and cGVHD). In addition, whereas irreversible inhibitors (particularly the most used, Ibrutinib) lose potency against Cys481 mutants (such as C481R, T474I, and T474M), some noncovalent inhibitors maintain inhibition against BTK variants, providing a potentially effective treatment option for Ibrutinib-resistant or naïve patients [22].

To overcome resistance problems, different and innovative medicinal chemistry strategies are represented by: (i) PROTAC molecules; (ii) a multi-target approach with compounds able to simultaneously target BTK and FAK [35]; (iii) the association of classical BTKIs with other chemotherapeutics, antibodies, or immunotherapies with the aim to inhibit different signal pathways at intracellular levels; and (iv) the nanoformulations of BTKIs (including gold, polymeric NPs, and aqueous nanosuspension) that are able to also reduce toxicity and ameliorate absorption and bioavailability [7].

All these different approaches could represent valid help in overcoming resistance and the side effect onset of the irreversible BTKIs in clinical use.

## 8. Conclusions

In recent years, BTK has emerged as a new target in medicinal chemistry and many BTKIs have been patented and reported in the literature. To date, only five irreversible BTKIs have been launched on the market to treat different types of leukemia, lymphomas, and cGVHD, whereas reversible BTKIs are under clinical investigation for the treatment of autoimmune diseases, especially RA and MS. From 2020, some approved BTKIs (especially Acalarutinib) are under study for COVID-19 therapy.

For all these reasons, the search for new BTKIs is still of great interest to the academic community and pharmaceutical industry, representing a therapeutic priority in medicinal chemistry research.

## Figures and Tables

**Table 1:** Name, chemical structure and treated diseases of approved BTKIs.

Drug	Molecular Formula	Diseases
Ibrutinib	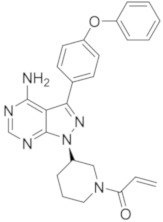	CLL/SLL WM MZL MCL cGVHD
Acalabrutinib	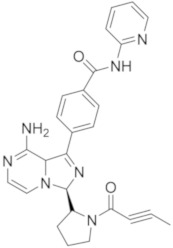	CLL SLL MCL
Zanubrutinib	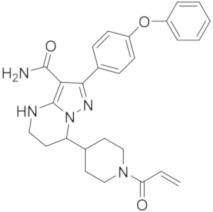	MCL
Tirabrutinib	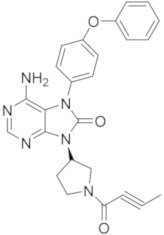	CNS lymphoma WM CLL
Orelabrutinib	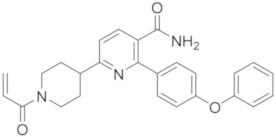	MCL, CLL SLL

**Table 4:** Chemical class of (ir)reversible BTKIs reported in literature from 2017 to date and corresponding references.

Chemical Class	Type of Inhibition	Ref
Diphenylaminopyrimidines	Irreversible BTKIs	[64,65,66,67,68,69,70,71,72,73,74,75,76]
Pyridines	Irreversible BTKIs	[77]
Pyrimidines	Reversible BTKIs	[78]
Pyridinones	Irreversible BTKIs	[79,80]
Triazines	Irreversible and reversible BTKIs	[81,82,83]
Pyrazolo-pyrimidines	Irreversible BTKIs	[84,85,86,87,88,89]
Thieno-pyrimidines	Irreversible BTKIs	[90,91,92]
Pyrrolo-pyrimidines	Irreversible BTKIs	[93,94,95,96,97,98]
Imidazo-pyrazines	Reversible BTKIs	[99,100,101]
Imidazo-pyridines	Reversible BTKIs	[102,103]
Imidazo-pyrazoles	Irreversible BTKIs	[104]
Quinolines	Irreversible and Reversible BTKIs	[105,106,107]
Dihydroisoquinolines	Irreversible BTKIs	[108]
Phthalazines	Reversible BTKIs	[109,110,111]
Carbazoles	Irreversible BTKIs	[112]
Tetrahydrocarbazole	Irreversible BTKIs	[112]
Indoles	Irreversible BTKIs	[112]
Pyrano-chromenone	Irreversible BTKIs	[113]
Thiazoles	Reversible BTKIs	[114]
Pyrazoles	Reversible BTKIs	[115,116]
benzofuranes	Irreversible BTKIs	[117]
PROTAC	Irreversible and reversible BTKIs	[11,12]

## Abbreviations

BCR	B-cell receptor
BTK	Bruton’s kinase
BTKIs	BTK inhibitors
CLL	lymphocytic leukemia
CIA	collagen-induced arthritis
DPPY	diphenylaminopyrimidine
EGFR	epidermal growth factor receptor
FAK	focal adgesion kinase
FBDD	fragment-based drug discovery
cGVHD	chronic graft versus host disease
MZL	marginal zone lymphoma
MCL	mantle cell lymphoma
MS	multiple scherosis
NSCLC	non-small cell lung cancer
PMDA	Pharmaceuticals and Medical Devices Agency
PH	pleckstrin homology domain
Pho-DPYYs	diphenylpyrimidine derivatives
PLCγ2	phospholipase γ2
PK	pharmacokinetic
PROTAC	proteolysis-targeting chimera compounds
PRR	proline-rich regions
RA	rheumatoid arthritis
RMS	Relapsing Multiple Sclerosis
ROS	reactive oxygen species
SBD	structure-based design
SLL	small lymphocytic lymphoma
SH2/SH3	Src homology 2/3 domain
SLE	Systemic Lupus Erythematosus
TK	tyrosine kinases
WM	Waldenström’s macroglobulinemia
WT	wild type
